# Implication of the Transmembrane Domain in the Interleukin 10 Receptor Platform Oligomerisation

**DOI:** 10.3390/cells12101361

**Published:** 2023-05-10

**Authors:** Thomas Kuntzel, Caroline Spenlé, Lucas D. Pham-Van, Dafni Birmpili, Aurélien Riou, Aurore Loeuillet, Imane Charmarke-Askar, Dominique Bagnard

**Affiliations:** 1 UMR7242 Biotechnology and Cell Signalling, Centre National de la Recherche Scientifique, Strasbourg Drug Discovery and Development Institute (IMS), University of Strasbourg, 67400 Illkirch-Graffenstaden, France; 2 Ecole Supérieure de Biotechnologie de Strasbourg, 67400 Illkirch-Graffenstaden, France

**Keywords:** transmembrane domain, interleukin 10, peptide, macrophage

## Abstract

Interleukin 10 (IL-10) exerts anti-inflammatory and immune regulatory roles through its fixation to the IL-10 receptor (IL-10R). The two subunits (IL-10Rα and IL-10Rβ) organise themselves to form a hetero-tetramer to induce the activation of the transcription factor STAT3. We analysed the activation patterns of the IL-10R, especially the contribution of the transmembrane (TM) domain of the IL-10Rα and IL-10Rβ subunits, as evidence accumulates that this short domain has tremendous implications in receptor oligomerisation and activation. We also addressed whether targeting the TM domain of IL-10R with peptides mimicking the TM sequences of the subunits translates into biological consequences. The results illustrate the involvement of the TM domains from both subunits in receptor activation and feature a distinctive amino acid crucial for the interaction. The TM peptide targeting approach also appears to be suitable for modulating the activation of the receptor through its action on the dimerization capabilities of the TM domains and thereby constitutes a potential new strategy for the modulation of the inflammation in pathologic contexts.

## 1. Introduction

The interleukin 10 (IL-10) receptor is composed of a heterodimer of two subunits: IL-10Rα, which is exclusive and has a high affinity for IL-10 and IL-10Rβ, which has low-affinity and is shared with other receptors [1]. IL-10 is secreted as a homodimer, interacting with two copies of the heterodimeric receptor complex [2,3]. These interactions have been demonstrated by cryogenic electron microscopy for the extracellular domain of the receptor [3,4]. For the transmembrane (TM) part of the receptor, however, no data show its implication in the oligomerisation of the subunits, nor the transduction of the signal. The TM domain of receptors has long been reduced to a simple membrane anchoring role. However, several studies show an active role in receptor oligomerisation and activation [5,6,7]. Consequently, the TM domain constitutes a therapeutic target for the modulation of the activation of the membrane receptors. In the case of the IL-10 receptor, such a modulation could have profound repercussions on the inflammatory status of the immune system. Indeed, after recognition of the ligand, the receptor induces the activation of intracellular kinases, leading to the activating phosphorylation of the transcription factor STAT3 [1,8]. This signalling is responsible for anti-inflammatory consequences on the cells, such as inhibition of the secretion of pro-inflammatory cytokines by monocytes, macrophages and CD4^+^ T cells. IL-10 signalling also favours survival, proliferation and activation of B cells and CD8^+^ T cells [9,10]. Among the roles attributed to IL-10, we focused on its effects on macrophage activation. These immune cells have the ability to endorse several phenotypes with opposite effects. The extremes of these phenotypes are called M1 for pro-inflammatory macrophages and M2 for anti-inflammatory ones [11]. This description is oversimplified and in reality, polarisation has a high plasticity and macrophages adopt a phenotype situated on a continuum between these extremes [12]. However, in pathological situations, circulating and resident macrophages are often implicated with a dysregulation of their polarisation state and an imbalance between the M1 and M2 markers of polarisation is described [13]. To define a phenotype for these cells, several markers are used depending on their effects. For the definition of M2-like polarisations, markers with anti-inflammatory activities are assessed. One of these markers is IL-10 and its receptor, known for its strong anti-inflammatory effect [14,15]. Targeting the polarisation state of macrophages could be beneficial in these pathological states by inhibiting the detrimental activities they display [16].

Here, we assessed for the first time the implication of the TM domain in IL-10R oligomerisation and determined if it can be targeted in order to interfere with its signalling. We then established if modifying the activation of IL-10R is translated into biological consequences.

## 2. Materials and Methods

### 2.1. In Silico Simulation Tools

For in silico simulations, two tools were used: PREDDIMER and PDBePISA. The first was developed by A.A. Polyansky and P.E. Volynsky in the R.G. Efremov lab [17]. It allows the prediction of the structure of dimeric transmembrane helices. From the TM sequences, it can reconstruct dimer structures, rank and filter them with a scoring function (FSCOR), generate three-dimensional structures, and display the hydrophobicity of the helices and their interacting interfaces on two dimensional maps [17]. The second tool, PDBePISA (Protein Data Bank of Europe, Proteins, Interfaces, Structures and Assemblies), was developed by E. Krissinel and K. Henrick [18]. It enables the exploration of macromolecule interfaces based on several physicochemical properties (free energy of complex formation, solvation energies, interaction surface, hydrogen bonds, hydrophobicity) and the determination of the buried surface area (BSA) of each amino acid [18].

### 2.2. Cell Culture

RAW264.7 and BV-2 cells were cultured in a 10 cm Petri dish filled with DMEM (Dulbecco’s Modified Eagle Medium) High glucose (4.5 g/L) with stable glutamine and sodium pyruvate (Dutscher, Bernolsheim, France), supplemented with 10% inactivated foetal bovine serum (FBS, Gibco, ThermoFisher Scientific, Waltham, MA, USA) and 1% penicillin-streptomycin (10,000 U/mL–10 mg/mL, PanBiotech, Aidenbach, Germany), in an incubator at 37 °C with 5% CO_2_.

HEK-293 cells were cultured in a 10 cm diameter Petri dish filled with DMEM high glucose (4.5 g/L) with stable glutamine and sodium pyruvate, supplemented with 10% inactivated foetal bovine serum, in an incubator at 37 °C with 5% CO_2_.

### 2.3. Peptides

Peptides were synthetized by the Peptide Specialty Laboratories GmbH (Heidelberg, Germany) by solid-phase peptide synthesis (Fmoc chemistry). The peptide corresponding to the TM sequence of IL-10Rα, KGDLVISMLLFCGILVCLVLQWYIRKR (in one-letter code), is referred to as MTP-IL-10Rα. The peptide corresponding to the TM sequence of IL-10Rβ, KGDAIILIVSVLVVFLFLLGRKR (in one-letter code), is referred to as MTP-IL-10Rβ. Peptide purity estimated by RP-HPLC was more than 90%, according to manufacturer’s indication. Peptide powders were solubilized at 10^−7^ M in dimethylsulfoxide (DMSO, Eurisotop, Saclay, France) for in vitro experiments.

### 2.4. BRET Assay

The sequences of the receptors to test were cloned into plasmids, which encoded either the Renilla luciferase Rluc (as a donor, plasmid pRL-CMV E2261, Promega, Madison, WI, USA) or the enhanced yellow fluorescent protein eYFP (as an acceptor, plasmid pEYFP-N1, 6006-1, Clontech, Mountain View, CA, USA). Plates for transfection were prepared using a Biomek FX^P^ pipetting robot (Beckman Coulter, New York, NY, USA). In each well of 96-well plates (Greiner, Kremsmünster, Austria), a combination of Rluc and eYFP-expressing plasmids, diluted to the appropriate concentration in 20 µL Tris-EDTA (TE) buffer (Sigma, St. Louis, MO, USA), was deposited with 8 µL CaCl_2_ (Sigma) and 26 µL BES (Sigma).

HEK293 cells were seeded in a 1/200 collagen I-coated (4 mg/mL, Sigma) 96-well plates (Corning, Lowell, MA, USA) at a density of 20,000 cells per well in DMEM high glucose supplemented with 10% inactivated FBS. All the following steps were performed using the FX^P^ pipetting robot. Twenty-four hours after seeding, cells were transfected with the transfection medium prepared upstream. After four hours, the transfection medium was removed, and cells were washed with PBS. DMEM high glucose (4.5 g/L) without sodium pyruvate and phenol red (Sigma), supplemented with 10% inactivated FBS and 1% L-glutamine (Sigma) was then added for 24 h. Afterwards, medium was removed, cells were washed again with PBS, and medium was replaced with HBSS (Hank’s Balanced Salt Solution, Sigma). Using a Biomek NX^P^ pipetting robot (Beckman Coulter), coelenterazine H (Interchim, Los Angeles, CA, USA) at a final concentration of 5 µM was added. Finally, BRET signal was assessed by measuring bioluminescent and fluorescent signals at 37 °C at 485 ± 10 nm and 535 ± 12 nm, respectively, using a Victor Light (Perkin, Eden Praire, MN, USA) reader. BRET ratio was calculated using the following formula:

$$\mathrm{BRET}\;\mathrm{ratio}=\left(\frac{\mathrm{Fluorescence}\;\mathrm{signal}\;{\left(\mathrm{eYFP}\right)}_{\mathrm{test}}}{\mathrm{Bioluminescence}\;\mathrm{signal}\;{\left(\mathrm{Rluc}\right)}_{\mathrm{test}}}-\frac{\mathrm{Fluorescence}\;\mathrm{signal}\;{\left(\mathrm{eYFP}\right)}_{\mathrm{ctrl}\;\mathrm{Rluc}}}{\mathrm{Bioluminescence}\;\mathrm{signal}\;{\left(\mathrm{Rluc}\right)}_{\mathrm{ctrl}\;\mathrm{Rluc}}}\right)\times 1000.\;$$

where “test” corresponds to the co-transfected condition and “ctrl Rluc” corresponds to the Rluc transfected alone condition (background signal) [19].

Final BRET ratio is expressed in milliBRET units (mBu). BRET_max_ corresponds to the maximal BRET ratio recorded in mBu and BRET_50_ corresponds to 50% of the BRET_max_ signal. Fluorescence was also assessed with an EnVision Multimode plate reader (Perkin Elmer) at 535 nm after excitation at 485 nm to control transfection efficacy. For BRET saturation assays, the concentration of Rluc plasmid remains the same in each condition, whereas an increasing concentration of eYFP plasmid is added. If the BRET ratio curves obtained are not linear, with a R^2^ > 0.70, the signal obtained is specific. On the contrary, if the curve is linear, the result obtained is not specific, and corresponds to BRET of collision.

### 2.5. MTT Assay

The potential toxicity of MTP peptides was assessed using an MTT (3-(4,5-dimethylthiazol-2-yl)-2,5-diphenyl tetrazolium bromide) assay. RAW264.7 or BV-2 cells were seeded at 5000 cells per well in 100 μL of medium in a 96-well plate. The conditions tested were DMSO at a concentration of 0.5% and each peptide, at a concentration of 10^−7^ M. The plate was then incubated at 37 °C for the desired exposure time (4 h or 24 h). A 5 mg/mL MTT solution (Sigma) was diluted 1/50 with GBSS (Gey’s Balanced Salt Solution, Sigma). After removing the medium from each well, 100 μL of this MTT-GBSS solution was added per well. The plate was then incubated for 4 h at 37 °C. Then, 100 μL of isopropanol (VWR chemicals) was added to dissolve the purple crystals formed by the MTT reagent. The mixture in each well was aspirated and refilled to allow complete dissolution of the crystals. Finally, the optical density of each well of the plate is read by spectrophotometry (MultiskanGo, ThermoFisher Scientific) at 570 nm.

### 2.6. Proximity Ligation Assay

For proximity ligation assay (Duo-Link), RAW264.7 or BV-2 cells were seeded at 20,000 cells per well on Lab-Tek Permanox slides overnight and then treated with the appropriate peptide at 10^−7^ M for one hour. After fixation with 2% PFA for 10 min, cells were permeabilized with PBS/0.1% Triton X-100 for 10 min. For heterodimer detection, primary antibodies (IL-10Ra OAAF01119, Aviva, San Diego, CA, USA and IL-10Rb AF5368, R&D, Minneapolis, MN, USA) were incubated together by pair overnight at 4 °C, diluted at 1/100 in PBS. The next day, the secondary antibodies (PLA probe anti goat MINUS 5X and PLA probe anti rabbit PLUS 5X, Sigma), diluted 1/5 in PBS, were added for one hour at 37 °C. The proximity ligation assay was then performed according to the manufacturer’s recommendations with the “detection orange” kit (Sigma). Quantification of the dots was performed using ImageJ software.

### 2.7. Western Blot

For the assessment of STAT3 phosphorylation, RAW264.7 or BV-2 cells were seeded in 6-well plates and treated for 1 h with the appropriate peptide and 15 min with 100 ng/mL IL-10 (Miltenyi, Bergisch Gladbach, Germany). Proteins were extracted using PBS-Triton 1%, proteinase inhibitors and phosphatases inhibitors (Sigma). Proteins were separated on a 4–20% SDS-PAGE gel (sodium dodecyl sulfate—polyacrylamide gel electrophoresis) and transferred onto a nitrocellulose membrane (Trans-Blot Turbo System, BioRad, Hercules, CA, USA). The blots were soaked in blocking solution (Tris-Buffered Saline (TBS)), 0.1% Tween-20, BSA (bovine serum albumin, 5%) for 30 min at room temperature. Primary antibody Anti-STAT3 antibody (ab119352) diluted 1/1000 or Anti-STAT3 (phospho Y705) antibody (ab76315) diluted 1/2000 was incubated overnight at 4 °C in TBS, 0.1% Tween-20, BSA 5%. After several washes, secondary antibody (Goat anti-mouse IgG HRP (HorseRadish Peroxidase) conjugate for STAT3 and Goat anti-rabbit IgG HRP conjugate for Anti-STAT3, Bio-Rad, 1/3000) was incubated for 1 h at room temperature. The revelation step was performed using Clarity-Enhanced Chemiluminescence Blotting Substrates (Bio-Rad, Hercules, CA, USA) according to the manufacturer’s instructions. Images of the immunoblots were acquired and analysed using the Chemidoc Touch Imaging System (Bio-Rad) and normalized with the stain-free method.

### 2.8. Statistics

All statistical analyses were performed using GraphPad Prism 9.0 (GraphPad Software) and presented as mean ± standard error of the mean (SEM). Parametric data were analysed using Student’s *t*-test for two groups and one-way ANOVA followed by Dunnett’s post hoc test for multiple comparisons to the control. Nonparametric data were analysed using the Kruskal–Wallis test.

## 3. Results

### 3.1. Prediction of the Interactions of the IL-10 Receptor Subunits

**Figure 1:**
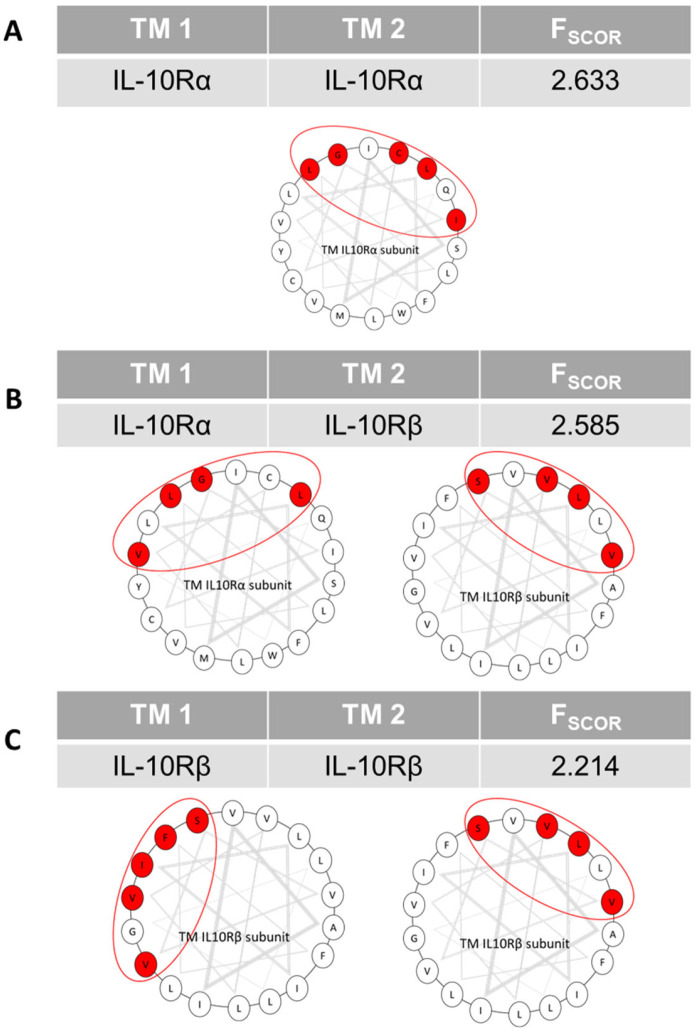
Predictions of IL-10R TM subunits interactions using PREDDIMER software. For each pair of TM sequences, this figure shows the PREDDIMER-calculated FSCOR and helical top views of the sequences. The grey triangles correspond to a helical turn of three amino acids. The circled areas represent the interaction interfaces. The amino acids in red are those implicated in the interaction, with the buried surface area values greater than or equal to 60%. (**A**) Prediction for IL-10Rα TM domain homodimerization. (**B**) Prediction for IL-10Rα/IL-10Rβ TM domain heterodimerization. (**C**) Prediction for IL-10Rβ TM domain homodimerization.

The interactions of the transmembrane domains of the subunits of the IL-10 receptor are poorly described. The implication of this receptor in inflammation and its role in pathological states led us to study its transmembrane domain, as it could be an interesting therapeutic target. We generated three-dimensional interacting models using the PREDDIMER software. From previous work in the lab, we had determined that an F_scor_ value above 2.6 is indicative for real biological interactions. The results in Figure 1 show that the TM sequences of the receptor have similar interaction scores for the IL-10Rα homodimer (Figure 1A) and for the heterodimer with IL-10Rβ (Figure 1B), whereas the score is weaker for the IL-10Rβ homodimer (Figure 1C). According to these results, the most probable TM oligomers are IL-10Rα homodimers and IL-10Rα/IL-10Rβ heterodimers. Afterwards, we determined the buried surface area (BSA) for each amino acid of the TM sequences of the subunits interacting, to identify the amino acids highly involved in the interaction. From these results we predicted for the IL-10Rα subunit the interaction interfaces involving the amino acids L_248_, G_251_, I_252_, C_255_ and L_258_ (LxxGIxxCxxL interface) for the homodimerization (Figure 1A) and L_248_, G_251_, V_254_ and L_258_ (LxxGxxVxxxL interface) for the heterodimerization with IL-10Rβ (Figure 1B). Strikingly, the predicted homodimerization motif of IL-10Rα is 60% similar to the motif for the heterodimerization with IL-10Rβ (Figure 1B). In consequence, IL-10Rα can form only one interaction at a time. For the IL-10Rβ subunit, homodimerization is possible through two interfaces, sharing only the amino acid S_230_. The first involves the amino acids I_226_, V_229_, S_230_, V_233_ and F_237_ (IxxVSxxVxxxF interface, Figure 1C) and the second L_227_, S_230_, V_231_ and V_234_ (LxxSVxxV interface, Figure 1C). The second interface is 100% similar to the interface used for the heterodimerization with IL-10Rα (Figure 1B). According to these results, IL-10Rβ is capable of forming both heterodimers and homodimers simultaneously. The results of the predictions indicate that the TM domain of IL-10Rα interacts either with another IL-10Rα TM domain or with an IL-10Rβ TM domain. IL-10Rβ can interact with two subunits at the same time but is more likely to form heterodimers.

### 3.2. Biological Validation of Predicted Interactions

**Figure 2:**
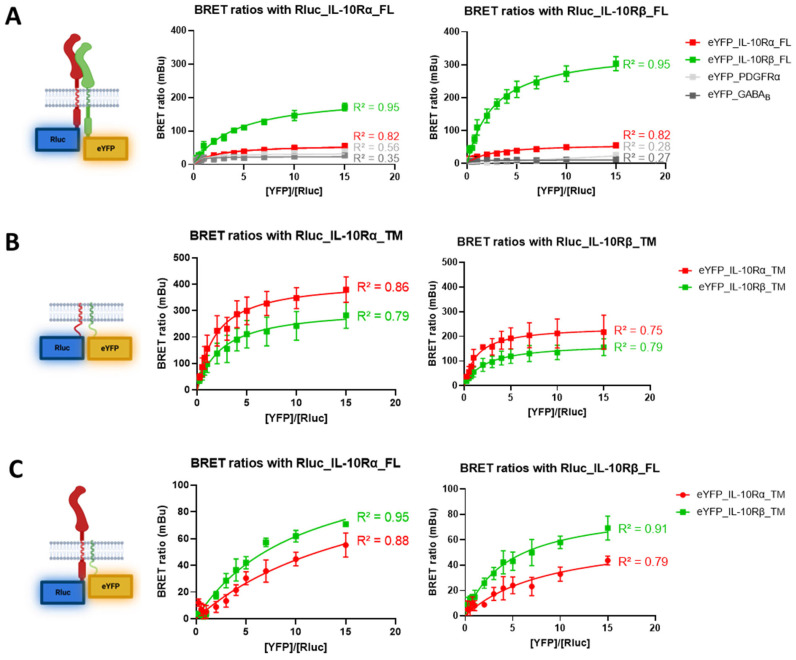
BRET validation of full length (FL) and transmembrane (TM) IL-10R subunits interactions. BRET saturation assays were performed to monitor the interaction of different pairs of proteins. The concentration of Rluc plasmid was kept constant for each condition (0.75 ng/mL), while the concentration of eYFP plasmid was increased in each condition. (**A**) BRET assay for full length receptors. Left graph shows the interactions of IL-10Rα with IL-10Rβ (N = 8), IL-10Rα (N = 8), PDGFRα (N = 2) and GABAB (N = 2). Right graph shows the interactions of IL-10Rβ with IL-10Rβ (N = 8), IL-10Rα (N = 8), PDGFRα (N = 2) and GABAB (N = 2). (**B**) BRET assay for TM sequences of the receptors. Left graph shows the interactions of IL-10Rα_TM with IL-10Rβ_TM (N = 6) and IL-10Rα_TM (N = 6). Right graph shows the interactions of IL-10Rβ_TM with IL-10Rβ_TM (N = 6) and IL-10Rα_TM (N = 6). (**C**) BRET assay for interactions of full-length receptors and TM sequences. Left graph shows the interactions of IL-10Rα_FL with IL-10Rβ_TM (N = 4) and IL-10Rα_TM (N = 4). Right graph shows the interactions of IL-10Rβ_FL with IL-10Rβ_TM (N = 4) and IL-10Rα_TM (N = 4). R^2^ are the coefficients of determination of the one site-specific binding non-linear fit curves.

To validate the biological relevance of the predicted interactions of the IL-10 receptor subunits, we performed a BRET saturation assay. In this assay, the first protein is tagged with an Rluc donor and a second with an eYFP acceptor. If the two tags are close enough, resonance energy transfer can occur, and a fluorescent signal emitted by the eYFP can be measured at 535nm. The BRET ratio determined afterwards exemplifies the interaction of the two proteins, as shown previously [19]. We first evaluated the BRET ratio for the interactions between the full-length subunits (Figure 2A).

For the IL-10Rα subunit, the interaction with itself yields a specific signal with a BRET_50_ value of 28 mBu (Figure 2A, left graph, red curve). The interaction with IL-10Rβ is also specific, and the BRET_50_ value reaches 86 mBu (Figure 2A, left graph, green curve). The test with two unrelated proteins (PDGFRα and GABA_B_) provides non-specific signals and serves as a negative control, where no interaction is measured. For the IL-10Rβ subunit, the homodimer reaches a BRET_50_ value of 152 mBu (Figure 2A, right graph, green curve), and the heterodimer a BRET_50_ of 28 mBu (Figure 2A, right graph, red curve). Here, again, non-specific signals were recorded when measuring the interaction with PDGFRα or GABA_B_. As reported by this experiment, the IL-10Rα subunit has a three-fold higher propensity to form heterodimers (+207% of the BRET_50_) rather than homodimers. However, IL-10Rβ has a five-fold stronger propensity to form homodimers (+443% of the BRET_50_ compared to homodimers).

In a second experiment, we assessed the interactions of the TM sequences only of the subunits (Figure 2B). For the TM domain of IL-10Rα, the BRET_50_ value is 190 mBu for the homodimer (Figure 2B, left graph, red curve) and reaches 141 mBu for the heterodimerization with IL-10Rβ (Figure 2B, left graph, green curve). The TM domain of IL-10Rβ showed a BRET_50_ of 78 mBu for the homodimer (Figure 2B, right graph, green curve) and 109 mBu for the heterodimer (Figure 2B, right graph, red curve). Thus, the TM segment interactions slightly differ from the whole protein as they are exhibiting a higher BRET_50_ for IL-10Rα homodimers (+35% compared to heterodimers) and for IL-10Rβ to form heterodimers (+40% compared to homodimers).

In a third experiment, we determined the interactions of the full-length Rluc-tagged receptors with only the eYFP-tagged TM sequences (Figure 2C). For the full-length IL-10Rα subunit, the specific interaction with the TM sequence of IL-10Rα yields a BRET_50_ value of 28 mBu (Figure 2C, left graph, red curve), which is the same value as for the full-length homodimer. The interaction with the IL-10Rβ TM domain reaches a value of 35 mBu (Figure 2C, left graph, green curve), which is 59% weaker than for the full-length heterodimer. For the full-length IL-10Rβ subunit, the BRET_50_ value for the interaction with the TM domain of IL-10Rβ is 35 mBu (Figure 2C, right graph, green curve), 77% lower than for the full-length homodimer. Lastly, the interaction with the TM domain of IL-10Rα reaches a value of 22 mBu (Figure 2C, right graph, red curve), which is 21% less than for the full-length heterodimer. These results indicate for the first time that the TM domains of the subunits of the IL-10R interact specifically and with variable intensities and thereby contribute to the dimerization of the whole receptor complex.

**Figure 3:**
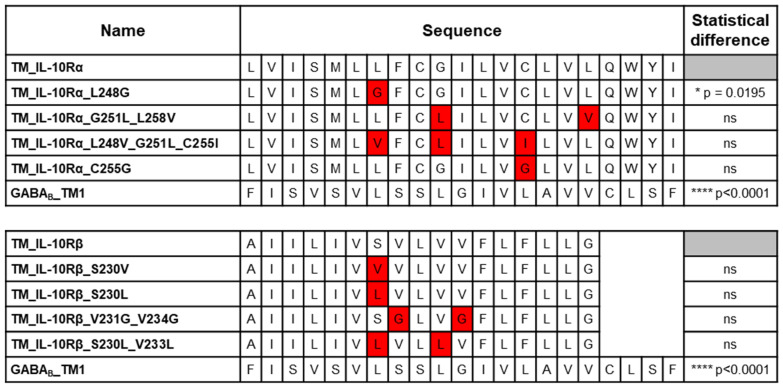
BRET evaluation of the interaction between the wild-type (WT) and the mutant TM sequences of the receptor subunits. BRET saturation assays were performed to monitor the interaction of different pairs of proteins. The concentration of Rluc plasmid was kept constant for each condition (0.75 ng/mL), while the concentration of the eYFP plasmid was increased in each condition. The sequences of the constructs tested are displayed in the second column. The mutations in the sequences are highlighted in red. The third column shows the statistical significance of the difference of BRET50 compared to the WT interaction. N = 4 for each interaction measured. ns: non-significant; **** *p* < 0.0001; * *p* = 0.0195; two-tailed parametric unpaired *t*-test.

Hence, we generated mutated TM sequences of the subunits by mutating the amino acids previously predicted as implicated in the interaction. Of all the generated point mutations, only one induced a significant reduction of the BRET signal. The substitution of leucine 248 by a glycine in the TM sequence of IL-10Rα leads to a decrease of 27% (*p* = 0.0195) of the BRET_50_ for the homodimerization with the wild-type TM sequence (Figure 3). This mutation only leads to a partial loss of the dimerization as compared with the level of interaction obtained with the non-related GABA_B_ TM1 sequence (−83%, *p* < 0.0001). These results identify leucine 248 of the IL-10Rα TM sequence as essential for full homodimerization. However, the other mutations were not sufficient to induce the loss of dimerization.

### 3.3. The TM Peptides Mimicking IL-10R Have No Toxic Effect on Cells

**Figure 4:**
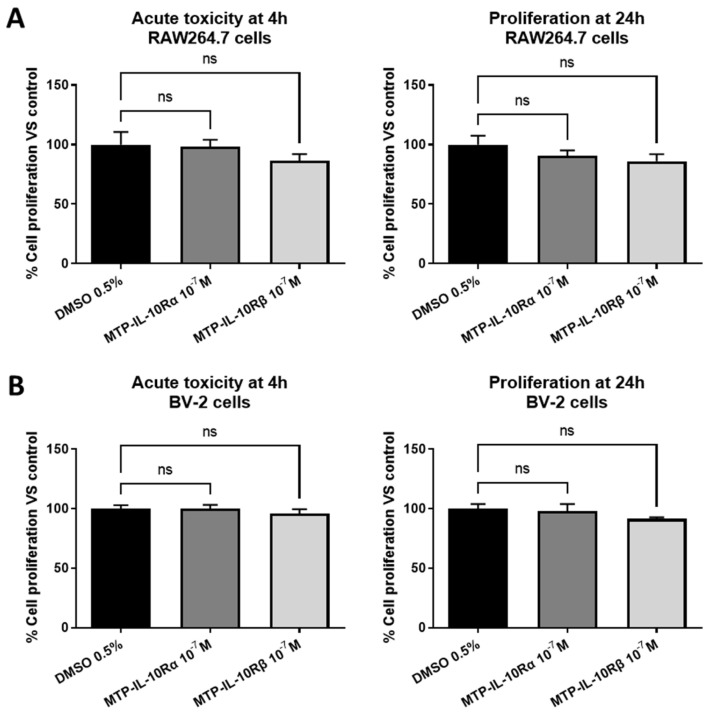
MTT assay for peptides. MTT assays, performed to assay peptides’ acute toxicity (4 h assay) and antiproliferative effect (24 h assay). RAW264.7 or BV2 cells were exposed to a 10^−7^ M concentration of MTP-IL-10Rα or MTP-IL-10Rβ peptides during 4 or 24 h, before measuring cell metabolic activity with tetrazolium dye. (**A**) MTT assay on RAW264.7 cells at 4 (left graph) or 24 h (right graph). (**B**) MTT assay on BV2 cells at 4 (left graph) or 24 h (right graph). ns: non-significant, Kruskal–Wallis test. Data are presented as mean ± SEM. N = 3 for each condition.

To determine the implication of the TM domains of the IL-10R subunits in the activity of the receptor, we designed two peptides, mimicking the TM sequences of IL-10Rα (MTP-IL-10Rα) and IL-10Rβ (MTP-IL-10Rβ). First, to assess the innocuity of the two peptides, we performed a MTT assay on two cells lines: RAW264.7 macrophages and BV-2 microglial cells. As presented in Figure 4, MTP-IL-10Rα and MTP-IL-10Rβ at 10^−7^ M display no acute toxic effect after four hours of exposition (left graphs), on the two cell lines. Similarly, the peptides do not display an antiproliferative effect after a 24 h exposition period (right graphs). These results indicate that the effects of the peptides observed in vitro are due to peptide activity and not toxicity or antiproliferative effects.

### 3.4. The TM Peptides Target IL-10R and Modulate Its Activation

For the assessment of the biological activities of the peptides, we performed in vitro assays on the two murine cell lines RAW264.7 macrophages and BV-2 microglial cells.

**Figure 5:**
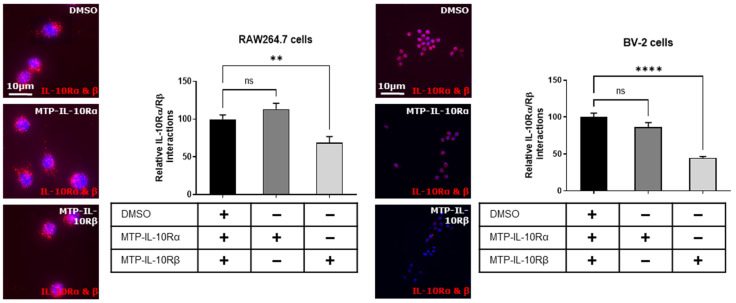
Proximity ligation assay for IL-10R subunits heterodimerisation in presence of peptides. Proximity ligation assay was performed on RAW264.7 and BV2 cells exposed to DMSO or DMSO-diluted peptides for one hour. In this assay, the amount of IL-10Rα/IL-10Rβ heterodimers was assessed in three different conditions: DMSO 0.1%, MTP-IL-10Rα (10^−7^ M) and MTP-IL-10Rβ (10^−7^ M). Representative images obtained by fluorescent microscopy are presented on the left for each cell type. DAPI staining is presented in blue. Each red dot corresponds to a hetero-interaction of the two subunits of the receptor. **** *p* < 0.0001; ** *p* < 0.01; ns: non-significant; one-way ANOVA followed by Dunnett’s post hoc test for multiple comparisons. Data are presented as mean ± SEM. N = 3 for each condition.

In a first step, we conducted a proximity ligation assay to evaluate the targeting capability of the peptides, and their effect on the heterodimerization of the subunits. On the RAW264.7 cells, we showed that MTP-IL-10Rα does not impair the heterodimerization of the two subunits (Figure 5A). Conversely, MTP-IL-10Rβ significantly inhibits the heterodimerization of the two subunits (−31%, *p* = 0.0045, Figure 5A). On the BV-2 cells, similar results were obtained, where MTP-IL-10Rα does not impair the heterodimerization of the two subunits and MTP-IL-10Rβ significantly inhibits the heterodimerization (−55%, *p* < 0.0001, Figure 5B).

**Figure 6:**
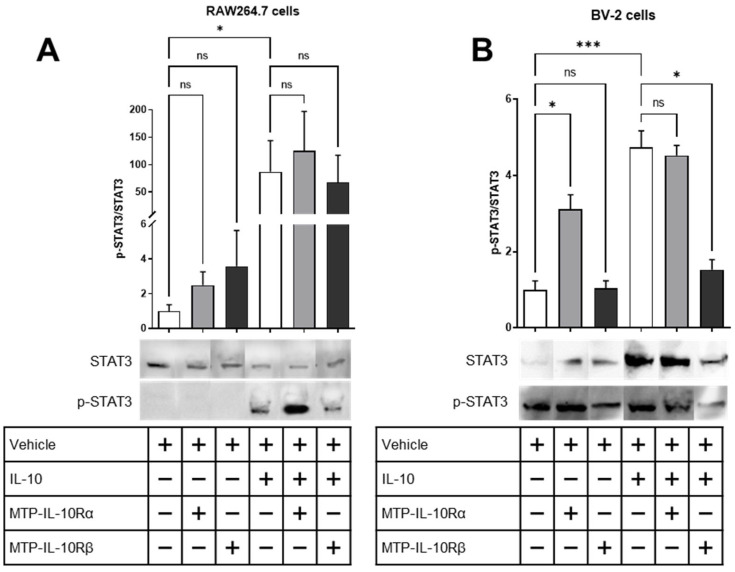
Measure of STAT3 phosphorylation in presence of IL-10 and peptides. Assessment of STAT3 phosphorylation by Western blot on lysates of RAW264.7 or BV2 cells exposed to IL-10 and/or DMSO or DMSO-diluted peptides. Graphs show the p-STAT3/STAT3 ratio. (**A**) p-STAT3/STAT3 ratio for the lysates of RAW264.7 cells exposed to DMSO 0.1% ± IL-10 (10 ng/mL), MTP-IL-10Rα (10^−7^ M) ± IL-10 or MTP-IL-10Rβ (10^−7^ M) ± IL-10. Photographs of the representative blots are presented on the right. N = 4. (**B**) p-STAT3/STAT3 ratio for the lysates of BV2 cells exposed to DMSO 0.1% ± IL-10 (10 ng/mL), MTP-IL-10Rα (10^−7^ M) ± IL-10 or MTP-IL-10Rβ (10^−7^ M) ± IL-10. Photographs of the representative blots are presented on the right. Full blots are presented in Figure S1. N = 5. *** *p* < 0.001; * *p* < 0.05; ns: non-significant; Kruskal–Wallis test. Data are presented as mean ± SEM.

In a second assay, we assessed the phosphorylation of STAT3, which is a downstream signal of IL-10R activation, in the presence of the IL-10 ligand or not. For the RAW264.7 cells, the peptides had no effect on the phosphorylation of STAT3 in the absence of IL-10 (Figure 6A). After treating the cells with 100 ng/mL IL-10 for 15 min, a significant increase in the phosphorylation of STAT3 is observed (87-fold increase, *p* = 0.0178, Figure 6A). However, after adding the peptides, no effect has been recorded. For the BV-2 cells, the peptide MTP-IL-10Rα is able to induce the phosphorylation of STAT3 in the absence of IL-10 (3-fold increase, *p* = 0.0443, Figure 6B), whereas MTP-IL-10Rβ is not. After treating the cells with IL-10, an increase in the phosphorylation of STAT3 has been measured (5-fold increase, *p* = 0.0005, Figure 6B). In this case, after adding the peptides, MTP-IL-10Rα had no effect, whereas MTP-IL-10Rβ was able to significantly inhibit the phosphorylation of STAT3 (−68%, *p* < 0.0119, Figure 6B). Hence, these results indicate that MTP-IL-10Rα is able to induce the activation of the signalling cascade of the IL-10R in absence of the ligand, but only in microglial cells. MTP-IL-10Rβ for its part, inhibits the IL-10 induced phosphorylation of STAT3 in both macrophages and microglia, which confirms the inhibition of heterodimerization observed by the proximity ligation assay.

## 4. Discussion

Interleukin 10 is a cytokine well-known for its anti-inflammatory and immune regulatory roles. After activation of its receptor, the downstream signalling cascade is implicated in the inhibition of pro-inflammatory cytokines secretion such as IL-1β, IL-6, IL-12, IFNγ and TNFα, with profound changes in the activities of the immune cells [20]. However, the mechanisms of the activation of the receptor platform are only described for the extra- and intracellular domains, although increasing evidence shows the importance of the TM domain in the oligomerisation and activation of various receptors, together with its druggability [5,6,7,21,22]. The aim of this study is to decipher the role of the TM domains of the two receptor subunits in its activity. First, we generated in silico models of the interactions between the TM domains of the two subunits and disclosed the amino acids involved in these interactions. According to these results, IL-10Rα TM forms homodimers and heterodimers with IL-10Rβ TM at an interface sharing 60% of the amino acids (L_248_, G_251_ and L_258_), meaning that IL-10Rα TM can only be involved in one interaction at a time. On the contrary, IL-10Rβ TM possesses two distinct interfaces, one forming only homodimers, the other forming homodimers as well as heterodimers with IL-10Rα. The only common amino acid between the two interfaces is serine 230. Thus, the TM domain of IL-10Rβ is able to form homodimers and heterodimers simultaneously. Interestingly, no classic interaction motif has been identified, in so far as there is no (G, A, S)xxx(G, A, S) motif, nor leucine or glycine zipper [5]. The most recent data about the interactions of the extracellular domains, obtained by cryogenic electron microscopy (Cryo-EM) by the Garcia lab, showed that after fixation of the ligand, IL-10Rα forms a heterodimer with IL-10Rβ, which is in accordance with our results [4]. An explanation for the second interface of IL-10Rβ is its recognition capability of other IL-10R family subunits (IL20Rα, IL22R and IL28R), whereas IL-10Rα is exclusive to IL-10 [1].

To validate these models, we performed a saturation BRET assay in vitro by tagging the full-length subunits or only the TM domains with the two reporters for this assay (Rluc and eYFP). The experiment allowed us to verify the specificity of the signal obtained. For the full-length interactions, specific signals were obtained for the interactions of the subunits of the receptor and not for non-related proteins (PDGFRα and GABA_B_). Concerning the interactions of the subunits, we noticed that if IL-10Rα is the donor, it has a threefold stronger propension to form heterodimers, which is consistent with the in silico and cryo-EM results. Surprisingly, if IL-10Rβ is the donor, it seems to have much lower efficiency in forming heterodimers with IL-10Rα. An explanation could be a steric hindrance in the assay resulting in a larger distance between the reporters, and a wrong orientation of their dipole moments [23]. Differences in these features result in an asymmetry of the BRET system which could be alleviated by performing other BRET experiments to verify its selectivity as competition experiments or by increasing the concentrations of the reporters at a constant acceptor/donor ratio [24]. Moreover, the results show that IL-10Rβ has a fivefold stronger propensity to constitute homodimers, which could be an explanation of the low signal for the heterodimer. This propensity can be related to the recent work of Mossner, Kuchner et al. showing that a homodimeric IL-10Rβ can transduce a signal with biological consequences initiated by the cytokine IL-22 [25]. In a second experiment, we demonstrated that the TM domains of the subunits also interact in a specific way. In that case, each TM subunit can form homodimers as well as heterodimers, with differences in the signal between both configurations lower than 40%. Afterwards, we combined both experiments and showed also that the full-length receptors interact specifically with the TM subunits. Interestingly, the results indicate that full-length IL-10Rα is more likely to form a heterodimer with TM IL-10Rβ, and full-length IL-10Rβ a homodimer with TM IL-10Rβ, as for the full-length interactions in the first BRET experiment. From these results, one can speculate that the TM domain is also involved in the correct oligomerisation of the IL-10R, in addition to the extra- and intracellular domains. However, the signals obtained from the last experiment are around 60–80% lower, which may be due to an important distance between the two reporters, because of an important difference in the size of the proteins. In the last BRET experiment, we generated mutated TM sequences, in order to determine the amino acids implicated in the interaction. Most of the mutations do not interfere with the interaction of the TM domains, except for a L248G mutation of the IL-10Rα subunit, which significantly diminishes the interaction (−27%, *p* = 0.0195). Because the interaction signals with a non-related sequence (GABAB_TM1) are weaker (−83%, *p* < 0.0001), our data suggest the existence of a specific interaction motif which has yet to be fully identified.

For a better characterization of the implication of this TM domain in the receptor’s oligomerisation, we conducted a series of in vitro tests, using peptides mimicking the TM domains of the subunits of the receptor (MTP-IL-10Rα and MTP-IL-10Rβ). We tested two peptides on two cell lines: RAW264.7 and BV-2 cells. The first corresponds to macrophage-like cells retrieved from a tumour in a male BALB/c mouse infected with the Abelson leukaemia virus [26]. The second features microglial cells, derived from raf/myc-immortalised murine neonatal microglia obtained in C57BL/6 female mice [27]. In a proximity ligation assay, we assessed the repercussions of the peptides on the heterodimerization of the subunits of the receptor. In both cell lines, the results follow the same trend, and we observed that MTP-IL-10Rβ is able to inhibit heterodimerization of IL-10Rα and IL-10Rβ, contrarily to MTP-IL-10Rα which has no effect on heterodimerization. These results were not due to a toxic nor an antiproliferative effect, as seen by a MTT assay. However, the effect was stronger in BV-2 cells than in RAW264.7 cells, suggesting a difference in sensitivity between the two cell lines. For a better understanding of the effects of the peptides, we measured their impact on the downstream signalling cascade of IL-10R, by assessing the activating phosphorylation of STAT3. Again, a difference between both cell lines was observed. In RAW264.7 cells, the peptides had no effects, whereas in BV-2 cells, they altered the phosphorylation of STAT3. In fact, MTP-IL-10Rα induced the phosphorylation in the absence of the IL-10 ligand and MTP-IL-10Rβ inhibited the IL-10-induced phosphorylation. These results indicate that (1) both peptides are able to modulate the activation of the IL-10R and (2) a difference in sensitivity between a macrophage-like and a microglia-like cell line is observed. This difference may be due to a difference in the expression of the subunits of the receptor in the two cell lines, which has to be assessed. However, the ability of the peptides to alter the strong anti-inflammatory IL-10 signalling cascade prompted us to make the hypothesis that they are able to modulate the activation status of the cells. Macrophages and microglia are characterised by different polarisation states, depending on their pro- or anti-inflammatory roles. In several diseases, an imbalance between both polarisation states is often observed. Targeting these inflammatory states to rebalance them constitutes an interesting opportunity for the development of new therapeutic approaches [13]. MTP-IL-10Rα, which induces the IL-10 anti-inflammatory signalling cascade, could have interesting therapeutic effects in inflammatory diseases by inducing an anti-inflammatory, immune regulatory phenotype of macrophages and microglia [16]. The second peptide (MTP-IL-10Rβ), by inhibiting the activation of the IL-10 receptor, promotes an inflammatory response that could be of interest in cancers, where the immune system is repressed [28]. In order to assess the potential activity of these peptides, it would be interesting to test them in animal models of inflammatory and cancerous diseases. This type of membrane-targeting peptides has already been tested in vitro and in vivo in previous works of the laboratory, showing that they reach their target and possess biological activities [29,30,31,32,33].

To conclude, our study is the first to unravel the implication of the TM domain in the interactions of the subunits of the IL-10R. We found that the TM sequences of IL-10Rα and IL-10Rβ interact with the full-length proteins in a specific manner, and that certain amino acids of the TM sequence are essential for these interactions. Peptides mimicking the TM sequences of the two subunits impair the activation of the receptor in vitro. The peptide reproducing the sequence of TM IL-10Rα induces the activation of the receptor, whereas the peptide mimicking TM IL-10Rβ inhibits its activation. A better understanding of the effects of the peptides on the inflammatory status of macrophages and microglia remains to be clarified, but they constitute a new approach for targeting the IL-10R platform.

## Data Availability

Not applicable.

## Supplementary Materials

The following supporting information can be downloaded at: https://www.mdpi.com/article/10.3390/cells12101361/s1, Figure S1: measure of STAT3 phosphorylation in presence of IL-10 and peptides: whole Western blots.
