# Supplementary material for: Implication of the Transmembrane Domain in the Interleukin 10 Receptor Platform Oligomerisation
Source: Cells. 2023 May 10;12(10):1361. doi: 10.3390/cells12101361 (PMC10216903; doi:10.3390/cells12101361)
Supplement: Supplementary file 1 [file cells-12-01361-s001.zip › cells-2309745-supplementary.pdf]

A

Immunoblot

STAT3 (88kDa)

Stain free

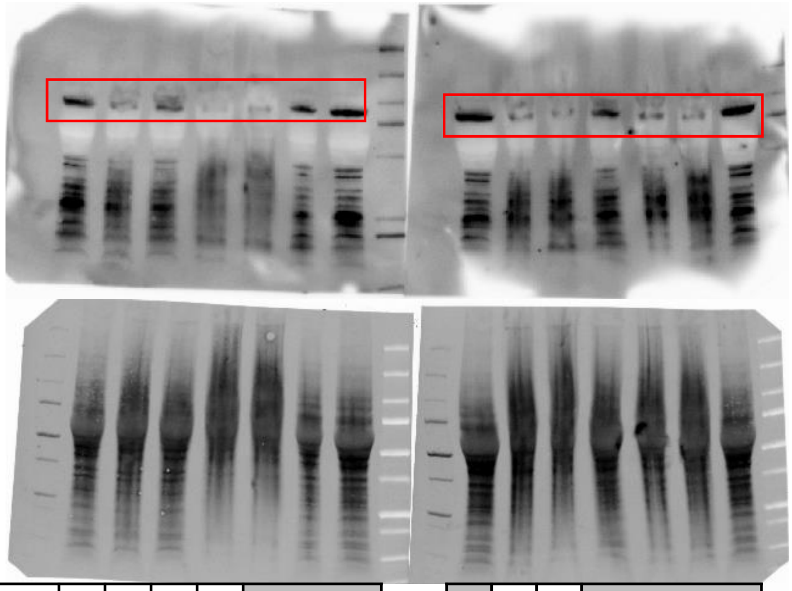

Control without peptide	+	-	-	-	Unrelated conditions
IL-10	-	+	-	+	
MTP-IL-10Rα	-	-	+	+	
MTP-IL-10Rβ	-	-	-	-	

	-	-	Unrelated conditions
	-	+	
	-	-	
	+	+	

Immunoblot

Phospho – STAT3 (88kDa)

Stain free

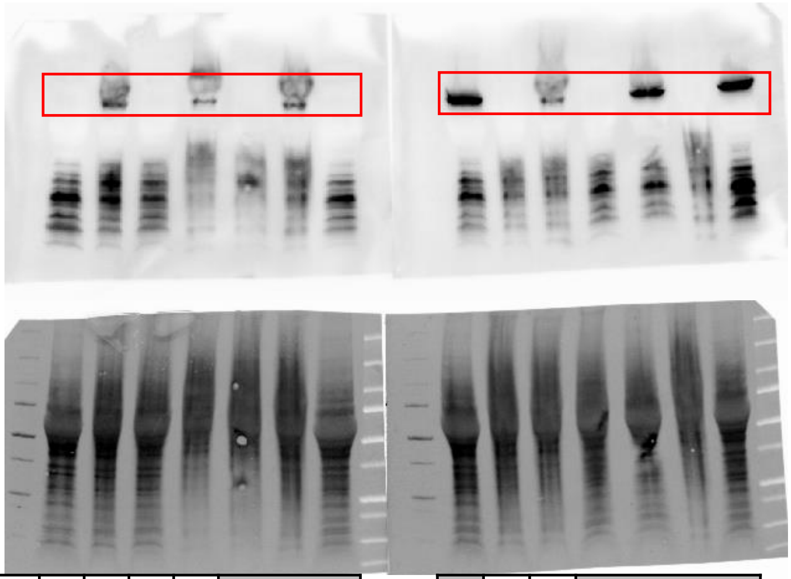

Control without peptide	+	-	-	-	Unrelated conditions
IL-10	-	+	-	+	
MTP-IL-10Rα	-	-	+	+	
MTP-IL-10Rβ	-	-	-	-	

	-	-	Unrelated conditions
	-	+	
	-	-	
	+	+	

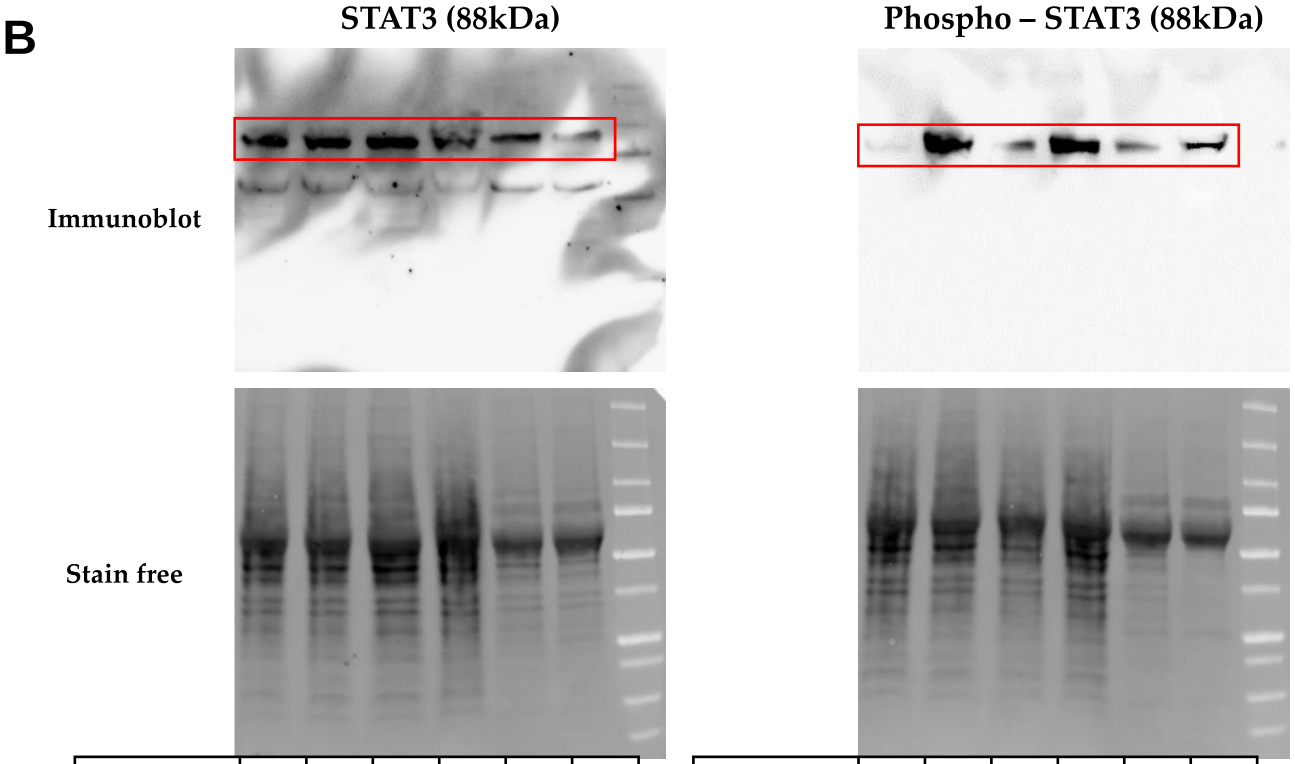

Vehicle	+	+	+	+	+	+
IL-10	-	+	-	+	-	+
MTP-IL-10Rα	-	-	+	+	-	-
MTP-IL-10Rβ	-	-	-	-	+	+

Vehicle	+	+	+	+	+	+
IL-10	-	+	-	+	-	+
MTP-IL-10Rα	-	-	+	+	-	-
MTP-IL-10Rβ	-	-	-	-	+	+

Supplementary Figure S1. Measure of STAT3 phosphorylation in presence of IL-10 and peptides: whole western blots. (**A**) RAW264.7 cells. (**B**) BV-2 cells.
